# Emergence of a new antibiotic resistance mechanism in India, Pakistan, and the UK: a molecular, biological, and epidemiological study

**DOI:** 10.1016/S1473-3099(10)70143-2

**Published:** 2010-09

**Authors:** Karthikeyan K Kumarasamy, Mark A Toleman, Timothy R Walsh, Jay Bagaria, Fafhana Butt, Ravikumar Balakrishnan, Uma Chaudhary, Michel Doumith, Christian G Giske, Seema Irfan, Padma Krishnan, Anil V Kumar, Sunil Maharjan, Shazad Mushtaq, Tabassum Noorie, David L Paterson, Andrew Pearson, Claire Perry, Rachel Pike, Bhargavi Rao, Ujjwayini Ray, Jayanta B Sarma, Madhu Sharma, Elizabeth Sheridan, Mandayam A Thirunarayan, Jane Turton, Supriya Upadhyay, Marina Warner, William Welfare, David M Livermore, Neil Woodford

**Affiliations:** a Department of Microbiology, Dr ALM PG IBMS, University of Madras, Chennai, India; b Department of Infection, Immunity and Biochemistry, School of Medicine, Cardiff University, Cardiff, UK; c Health Protection Agency Centre for Infections, London, UK; d Department of Microbiology, Shaukat Khanum Cancer Hospital, Lahore, Pakistan; e Department of Microbiology, Pandit B D Sharma PG Institute of Medical Sciences, Haryana, India; f Department of Clinical Microbiology, Karolinska University Hospital, Stockholm, Sweden; g Department of Pathology and Microbiology, The Aga Khan University, Karachi, Pakistan; h Department of Microbiology, Amrita Institute of Medical Sciences, Kerala, India; i University of Queensland Centre for Clinical Research, University of Brisbane, Herston, QLD, Australia; j Department of Microbiology, Apollo Gleneagles Hospital, Kolkata, India; k Department of Medical Microbiology, Northumbria Healthcare NHS Foundation Trust, Tyne and Wear, UK; l Department of Microbiology, Apollo Hospitals, Chennai, India; m Department of Microbiology, Institute of Medical Sciences, Banaras Hindu University, Varanasi, India

## Abstract

**Background:**

Gram-negative Enterobacteriaceae with resistance to carbapenem conferred by New Delhi metallo-β-lactamase 1 (NDM-1) are potentially a major global health problem. We investigated the prevalence of NDM-1, in multidrug-resistant Enterobacteriaceae in India, Pakistan, and the UK.

**Methods:**

Enterobacteriaceae isolates were studied from two major centres in India—Chennai (south India), Haryana (north India)—and those referred to the UK's national reference laboratory. Antibiotic susceptibilities were assessed, and the presence of the carbapenem resistance gene *bla*_NDM-1_ was established by PCR. Isolates were typed by pulsed-field gel electrophoresis of XbaI-restricted genomic DNA. Plasmids were analysed by S1 nuclease digestion and PCR typing. Case data for UK patients were reviewed for evidence of travel and recent admission to hospitals in India or Pakistan.

**Findings:**

We identified 44 isolates with NDM-1 in Chennai, 26 in Haryana, 37 in the UK, and 73 in other sites in India and Pakistan. NDM-1 was mostly found among *Escherichia coli* (36) and *Klebsiella pneumoniae* (111), which were highly resistant to all antibiotics except to tigecycline and colistin. *K pneumoniae* isolates from Haryana were clonal but NDM-1 producers from the UK and Chennai were clonally diverse. Most isolates carried the NDM-1 gene on plasmids: those from UK and Chennai were readily transferable whereas those from Haryana were not conjugative. Many of the UK NDM-1 positive patients had travelled to India or Pakistan within the past year, or had links with these countries.

**Interpretation:**

The potential of NDM-1 to be a worldwide public health problem is great, and co-ordinated international surveillance is needed.

**Funding:**

European Union, Wellcome Trust, and Wyeth.

## Introduction

Bacteria from clinical and non-clinical settings are becoming increasingly resistant to conventional antibiotics. 10 years ago, concern centred on Gram-positive bacteria, particularly meticillin-resistant *Staphylococcus aureus* and vancomycin-resistant *Enterococcus* spp. Now, however, clinical microbiologists increasingly agree that multidrug-resistant Gram-negative bacteria pose the greatest risk to public health. Not only is the increase in resistance of Gram-negative bacteria faster than in Gram-positive bacteria,^1^, ^2^ but also there are fewer new and developmental antibiotics active against Gram-negative bacteria^3^, ^4^, ^5^, ^6^ and drug development programmes seem insufficient to provide therapeutic cover in 10–20 years.^7^, ^8^, ^9^

The increase in resistance of Gram-negative bacteria is mainly due to mobile genes on plasmids that can readily spread through bacterial populations. Standardised plasmid typing methods are enhancing our understanding of the host ranges of these elements and their worldwide distribution.^10^, ^11^ Moreover, unprecedented human air travel and migration allow bacterial plasmids and clones to be transported rapidly between countries and continents.^12^, ^13^ Much of this dissemination is undetected, with resistant clones carried in the normal human flora and only becoming evident when they are the source of endogenous infections. The CTX-M-15 extended-spectrum β-lactamase (ESBL) encoded by *bla*_CTX-M-15_ was first reported in India in the mid-1990s.^14^, ^15^ The gene jumped from the chromosome of its natural hosts, *Kluyvera* spp, to plasmids that have subsequently spread widely,^10^, ^16^ establishing CTX-M-15 as the globally-dominant ESBL and the primary cause of acquired resistance to third-generation cephalosporins in Enterobacteriaceae.^17^, ^18^

Recent surveys have identified ESBLs in 70–90% of Enterobacteriaceae in India and; although these collections might be a biased sample, they do suggest a serious problem, making the widespread use of reserved antibiotics such as carbapenems necessary.^15^, ^19^ Rates of cephalosporin resistance are lower in other countries but the growing prevalence of ESBL producers is sufficient to drive a greater reliance on carbapenems. Consequently, there is selection pressure for carbapenem resistance in Enterobacteriaceae, and its emergence is a worldwide public health concern since there are few antibiotics in reserve beyond carbapenems.^20^ Already *Klebsiella pneumoniae* clones with KPC carbapenemase are a major problem in the USA, Greece, and Israel, and plasmids encoding the VIM metallo-carbapenemase have disseminated among *K pneumoniae* in Greece.^21^

We recently reported a new type of carbapenem resistance gene, designated *bla*_NDM-1_.^22^ A patient, repatriated to Sweden after admission to hospital in New Delhi, India, was colonised by *K pneumoniae* and *Escherichia coli* with *bla*_NDM-1_ on plasmids of varying size, which readily transferred between bacterial strains in vitro. We sought molecular, biological, and epidemiological data on New Delhi metallo-β-lactamase 1 (NDM-1) positive Enterobacteriaceae in India and Pakistan and investigated importation of the resistance gene into the UK by patients returning from the Indian subcontinent.

## Methods

### Bacterial isolates

Isolates of bacteria were identified from Chennai and Haryana in India. UK isolates were identified from referrals to the Antibiotic Resistance Monitoring and Reference Laboratory by UK microbiology laboratories between 2003 and 2009. We also identified isolates from other sites around Bangladesh, India, and Pakistan.

### Procedures

Bacteria were identified via the Phoenix automated phenotypic identification criteria (Becton Dickinson, Oxford, UK) or with API 20E strips (bioMerieux, Basingstoke, UK). Minimum inhibitory concentrations (MICs) and carbapenem resistance were established by microbroth dilution (Phoenix), British Society for Antimicrobial Chemotherapy (BSAC) agar dilution, or disc diffusion.

Modified Hodge (cloverleaf) test involving distorted carbapenem inhibition zones and imipenem-EDTA synergy tests by disc, or the MBL Etest (AB bioMerieux, Solna, Sweden) were used to screen for metallo-β-lactamase production.^23^ The presence of *bla*_NDM-1_ was established by PCR with specific primers targeting the gene.^22^ PCR and sequencing were used to identify other resistant genes (*bla*_CMY-4_ and *bla*_CTX-M-15_) carried by the bacterial isolates.

Conjugational transfer of antibiotic resistance to the laboratory strain *E coli* J53 was done on blood agar without selection. After 18 h, the mixed cultures were washed from the plates, suspended in saline, and plated onto MacConkey agar containing sodium azide (100 mg/L) and meropenem (2 mg/L). Transconjugants were confirmed to have *bla*_NDM-1_ by PCR analysis. Plasmids were subsequently isolated and typed on the basis of their origins of replication, as described by Carattoli and colleagues.^11^

Genomic DNA was prepared in agarose blocks and digested with the restriction enzyme XbaI (Roche Diagnostics, Mannheim, Germany). DNA fragments were separated by pulsed-field gel electrophoresis (PFGE) on a CHEF-DR III apparatus (Bio-Rad, Hercules, CA, USA) for 20 h at 6 V/cm at 14°C with an initial pulse time of 0·5 s and a final pulse time of 30 s. Dendrograms of strain relatedness were created with BioNumerics software.

Genomic DNA in agarose blocks was digested with the restriction enzyme S1 (Invitrogen, Abingdon, UK). DNA fragments were separated by PFGE as above. In-gel hybridisation was done with a *bla*_NDM-1_ probe labelled with ^32^P (Stratgene, Amsterdam, Netherlands) with a random-primer method.^22^ Plasmid DNA bands that hybridised with *bla*_NDM-1_ were cut from the gel, purified, and typed as described by Carattoli and colleauges.^11^

### Role of the funding source

The sponsor of the study had no role in study design, data collection, data analysis, data interpretation, or writing of the report. The corresponding author had full access to all the data in the study and had final responsibility for the decision to submit for publication.

## Results

From Chennai, 75 *E coli*, 60 *Klebsiella* spp, and six other Enterobacteriaceae resistant to carbapenems were isolated from 3521 (4%) Enterobacteriaceae analysed throughout 2009. Of these 141 carbapenem-resistant Enterobacteriaceae, 44 (19 *E coli*, 14 *K pneumoniae*, seven *Enterobacter cloacae*, two *Proteus* spp, one *Citrobacter freundii*, and one *Klebsiella oxytoca*) were NDM-1 positive (about 1% of all resistant isolates). During that same period, 47 carbapenem-resistant isolates (24%) of 198 from Haryana were identified; from these, 26 (13%) were positive for NDM-1, and all were *K pneumoniae*. The Indian isolates from Chennai and Haryana were primarily from community acquired urinary tract infections, pneumonia, and blood-stream infections. The age range was 4–66 years with a mean of 36 years (SD 20) and a female to male ratio of about two to one.

**Figure 1:**
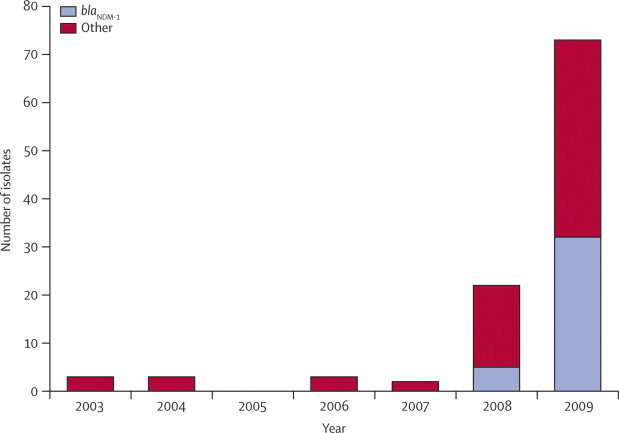
Numbers of carbapenemase-producing Enterobacteriaceae referred from UK laboratories to the UK Health Protection Agency's national reference laboratory from 2003 to 2009 The predominant gene is *bla*_NDM-1_, which was first identified in 2008. The other group includes diverse producers of KPC, OXA-48, IMP, and VIM enzymes.

In the UK resistant isolates increased in both 2008 and 2009 (figure 1). Isolates with the NDM-1 enzyme, which was first detected in the UK in 2008, became the predominant carbapenemase-producing Enterobacteriaceae in 2009, accounting for 32 (44%) of 73 carbapenemase producers. During 2008–09, 37 Enterobacteriaceae isolates with the NDM-1 enzyme were referred from 25 laboratories across England with single representatives also from Scotland and Northern Ireland. These were identified as *K pneumoniae* (21 isolates), *E coli* (seven), *Enterobacter* spp (five), *Citrobacter freundii* (two), *Morganella morganii* (one), and *Providencia* spp (one). They were from 29 patients and had been isolated from urine (15 patients), blood (three), burn or wound swab (four), sputum (two), central line tip (one), throat swab (one), or unknown specimens (three). The mean age of the patients was 60 years (SD 24; range 1–87), with 17 male patients and 12 female patients. At least 17 patients had a history of travelling to India or Pakistan within 1 year, and 14 of them had been admitted to a hospital in these countries. Reasons for these admissions included renal or bone marrow transplantation, dialysis, cerebral infarction, chronic obstructive pulmonary disease, pregnancy, burns, road traffic accidents, and cosmetic surgery.

Isolates, NDM-1-positive bacteria from Mumbai (32 isolates), Varanasi (13), and Guwahati (three) in India, and 25 isolates from eight cities in Pakistan (Charsadda, Faisalabad, Gujrat, Hafizabad, Karachi, Lahore, Rahim Yar Khan, and Sheikhupura) were also analysed in exactly the same manner but in laboratories in India and Pakistan. These isolates were from a range of infections including bacteraemia, ventilator-associated pneumonia, and community-acquired urinary tract infections.

**Figure 2:**
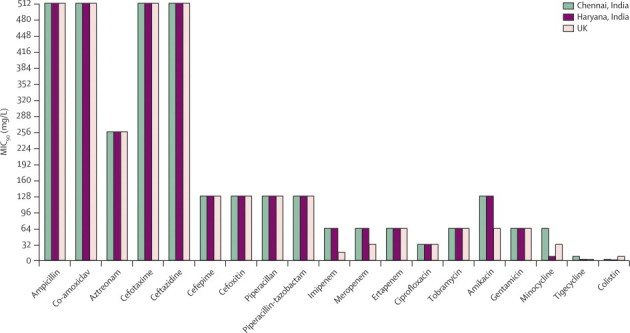
90% minimum inhibitory concentration (MIC_90_) for Enterobacteriaceae from Chennai and Haryana, India, and the UK

All the isolates producing the NDM-1 enzyme were resistant to several antibiotic classes (table). The 37 UK isolates were all resistant to imipenem and ertapenem, although a single *M morganii* isolate remained susceptible, at least in vitro, to meropenem (MIC 2 mg/L). Only four UK isolates remained susceptible to the monobactam aztreonam (MICs ≤1 mg/L), which is unaffected by metallo-carbapenemases including NDM-1; the other UK isolates were all resistant to all β-lactams, including aztreonam, suggesting the concurrent presence of additional β-lactamases including ESBLs and AmpC enzymes—identified by sequencing as mainly *bla*_CTX-M-15_ and *bla*_CMY-4_. All 37 isolates were resistant to amikacin and tobramycin, although one isolate was susceptible to gentamicin and three to ciprofloxacin. MICs of minocycline were consistently 2 mg/L or greater, interpreted as non-susceptible with the BSAC and European Committee on Antimicrobial Susceptibility Testing (EUCAST) breakpoints for doxycycline, but most (33 of 37) were susceptible to colistin (MICs ≤4 mg/L) and 26 were suseptible to tigecycline (MICs ≤1 mg/L; figure 2).

**Table:** Antibiotic susceptibilities for NDM-1-positive Enterobacteriaceae isolated in the UK and north (Chennai) and south India (Haryana)

	**UK (n=37)**		**Chennai (n=44)**		**Haryana (n=26)**	
	MIC_50_; MIC_90_ (mg/L)	Proportion susceptible*	MIC_50_; MIC_90_ (mg/L)	Proportion susceptible*	MIC_50_; MIC_90_ (mg/L)	Proportion susceptible*
Imipenem	32; 128	0%	64; 128	0%	32; 128	0%
Meropenem	32; 32	3%	32; >32	3%	>32; >32	3%
Piperacillin-tazobactam	>64; >64	0%	>64; >64	0%	>64; >64	0%
Cefotaxime	>256; >256	0%	>256; >256	0%	>256; >256	0%
Ceftazidime	>256; >256	0%	>256; >256	0%	>256; >256	0%
Cefpirome	>64; >64	0%	>64; >64	0%	>64; >64	0%
Aztreonam	>64; >64	11%	>64; >64	0%	>64; >64	8%
Ciprofloxacin	>8; >8	8%	>8; >8	8%	>8; >8	8%
Gentamicin	>32; >32	3%	>32; >32	3%	>32; >32	3%
Tobramycin	>32; >32	0%	>32; >32	0%	>32; >32	0%
Amikacin	>64; >64	0%	>64; >64	0%	>64; >64	0%
Minocycline	16; >32	0%	32; >32	0%	8; 16	0%
Tigecycline	1; 4	64%	4; 8	56%	1; 2	67%
Colistin	0·5; 8	89%†	1; 32	94%†	1; 2	100%†

MIC=minimum inhibitory concentration.

* Susceptibility defined by British Society for Antimicrobial Chemotherapy and European Committee on Antimicrobial Susceptibility Testing breakpoints; doxycycline breakpoints were used for minocycline.

† Colistin-resistant UK isolates were one isolate of *Morganella morganii* and one *Providencia* sp (both intrinsically-resistant species), also one *Klebsiella pneumoniae* and one *Enterobacter* sp.

The 44 isolates from Chennai were similarly resistant to all β-lactam antibiotics, fluoroquinolones, and aminoglycosides, apart from two that were sensitive to gentamicin. 39 were resistant to minocycline with MICs >2 mg/L, 19 to tigecycline, and three to colistin (table and figure 2). Two of the three isolates resistant to colistin were *Proteus* spp, which are intrinsically resistant, and the third was a *K pneumoniae* strain (colistin MIC >32 mg/L; tigecycline MIC 8 mg/L). Although several reports from Greece have noted *K pneumoniae* isolates as colistin resistant, we believe our isolate is truly pan-resistant.^24^, ^25^ Most of the 26 Haryana isolates were resistant to all β-lactam and non-β-lactam antibiotics, although three were susceptible to aztreonam and one to ciprofloxacin and amikacin. Minocycline MICs for the Haryana isolates were 8–16 mg/L and ten isolates had intermediate resistance (2 mg/L) to tigecycline by EUCAST criteria. None were resistant to colistin (table and figure 2).

The 21 *Klebsiella* isolates from the UK had different PFGE profiles and were typed to 19 distinct groups with only two related pairs, both of which included isolates from epidemiologically linked patients, probably representing cases of cross-infection. All the UK *E coli* isolates were different. The Chennai isolates were also very different, with none similar to each other. By contrast, the 26 NDM-positive *K pneumoniae* isolates from Haryana belonged to a single PFGE profile suggesting clonal spread. We could not prove statistically significant strain relatedness between the Indian and UK isolates.

**Figure 3:**
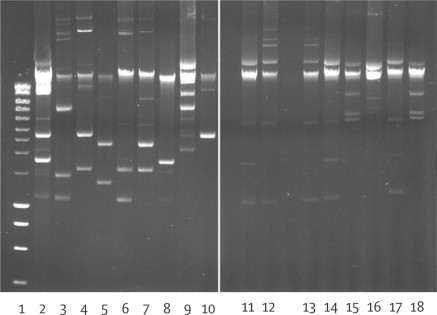
The difference in plasmid numbers from a selection of Indian isolates Tracks 1–10 show the number of plasmids in isolates from Chennai (south India) and tracks 11–18 show the number of plasmids in isolates from Haryana (north India). Most isolates contained up to seven plasmids, and in Chennai there was greater variation than in isolates from Haryana showing the bacterial clonality of NDM-1 carriage in Haryana.

Isolates from Chennai, Haryana, and the UK's Antibiotic Resistance Monitoring and Reference Laboratory were analysed for the location of the *bla*_NDM-1_ gene by S1 digestion of DNA, and then PFGE and direct probing of the gels with a radiolabelled *bla*_NDM-1_ gene. Each of the three groups of isolates typically carried several plasmids, with some isolates having up to eight plasmids (figure 3).

**Figure 4:**
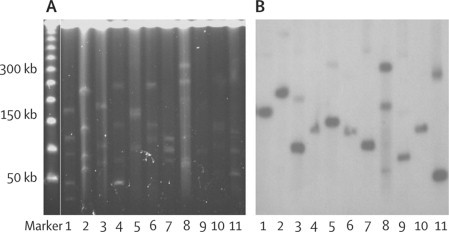
Hybridisation results of UK isolates with *bla*_NDM-1_ Pulsed-field gel of S1-treated plasmid DNA of UK isolates M15–M27 stained with ethidium bromide (A). Molecular weight marker is Lambda concatamer 50–1000 kb. The chromosome of each isolate is the bright band at the top of each lane and bright bands below are plasmids of various sizes. Autoradiogram of gel A probed with a *bla*_NDM-1_ showing individual or multiple plasmids in each strain carrying *bla*_NDM-1_ (B).

Indian isolates had *bla*_NDM-1_ exclusively on plasmids. Plasmids from the non-clonal Chennai isolates ranged from 50 kb to 350 kb, whereas those from the clonal *K pneumoniae* from Haryana were predominately either 118 kb (54%) or 50 kb (36%). The UK isolates had a more diverse range of plasmid sizes, 80 kb to greater than 500 kb. Three UK isolates also carried *bla*_NDM-1_ on their chromosome, suggesting in-situ movement of *bla*_NDM-1_. There were many plasmids of identical size in isolates collected from India and the UK (data not shown), suggesting plasmid movement between bacterial isolates. In some isolates, *bla*_NDM-1_ was carried on more than one plasmid (figure 4).

47 isolates from Chennai (33) and Haryana (14) were randomly chosen for further investigation with PCR and DNA probing to verify the origin of replication (incompatibility type) for plasmids carrying *bla*_NDM-1_.^11^, ^22^ Plasmids carrying *bla*_NDM-1_ from the 14 isolates from Haryana could not be typed. 13 of the 33 isolates from Chennai carried *bla*_NDM-1_ on A/C-type plasmids and one *bla*_NDM-1_ positive plasmid was incompatibility type FI/FII. Similarly, when the 32 randomly selected UK isolates were assessed with the same methods, 22 carried A/C type plasmids. The other *bla*_NDM-1_ positive plasmids from India and the UK that were A/C and FI/FII negative could not be typed.

Transconjugants were created in *E coli* J53 from the 33 Chennai and 32 UK isolates; however, the isolates from Haryana did not produce transconjugates.^22^ All transconjugants were shown by PCR to contain *bla*_NDM-1_. We compared the sizes of the plasmids in the clinical strains with those of the transconjugants and, in about 10% of cases, the plasmid had altered in size during transfer. In most cases the plasmid had lost DNA but two of 102 had gained DNA during transfer.

**Figure 5:**
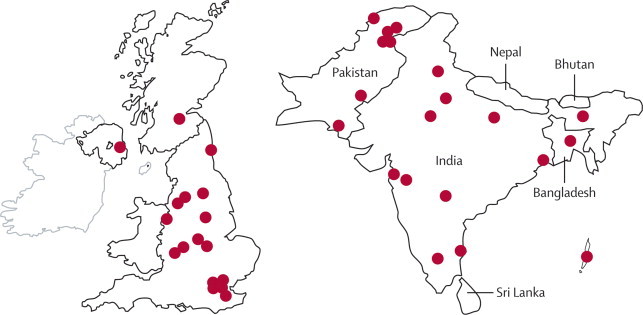
Distribution of NDM-1-producing Enterobacteriaceae strains in Bangladesh, Indian, Pakistan, and the UK

In addition to the collections of isolates from Chennai and Haryana detailed above, we have confirmed by PCR alone the presence of genes encoding NDM-1 in carbapenem-resistant Enterobacteriaceae isolated from Guwahati, Mumbai, Varanasi, Bangalore, Pune, Kolkata, Hyderabad, Port Blair, and Delhi in India, eight cities (Charsadda, Faisalabad, Gujrat, Hafizabad, Karachi, Lahore, Rahim Yar Khan, and Sheikhupura) in Pakistan, and Dhaka in Bangladesh (figure 5) suggesting widespread dissemination.

## Discussion

Enterobacteriaceae with NDM-1 carbapenemases are highly resistant to many antibiotic classes and potentially herald the end of treatment with β-lactams, fluoroquinolones, and aminoglycosides—the main antibiotic classes for the treatment of Gram-negative infections. Only a few isolates remained sensitive to individual aminoglycosides and aztreonam, perhaps owing to the loss of resistance genes (eg, those encoding aminoglycoside modifying enzymes, 16S rRNA methylases, or *bla*_CMY-4_).^12^, ^22^ Most isolates remained susceptible to colistin and tigecycline.

Typing did not identify common strain types of *E coli* or *K pneumoniae* between the Indian subcontinent and the UK or between north and south India. Nevertheless, the NDM-1-positive *K pneumoniae* isolates from Haryana were clonal, suggesting that some strains could potentially cause outbreaks. Most *bla*_NDM-1_ positive plasmids were readily transferable and prone to rearrangement, losing or (more rarely) gaining DNA on transfer. This transmissibility and plasticity implies an alarming potential to spread and diversify among bacterial populations. Curiously, many of the plasmids were incompatibility A/C types—a group not commonly associated with multidrug-resistant phenotypes.

Although antibiotic resistance in China has been highlighted as a concern,^4^ the rapid emergence of *bla*_NDM-1_ deserves equal attention. A recent editorial by Abdul Ghafur^26^ highlights the widespread non-prescription use of antibiotics in India, leading to huge selection pressure, and predicts that the NDM-1 problem is likely to get substantially worse in the foreseeable future. This scenario is of great concern because there are few new anti-Gram-negative antibiotics in the pharmaceutical pipeline and none that are active against NDM-1 producers.^20^ Even more disturbing is that most of the Indian isolates from Chennai and Haryana were from community-acquired infections, suggesting that *bla*_NDM-1_ is widespread in the environment.^27^

The introduction of NDM-1 into the UK is also very worrying and has prompted the release of a National Resistance Alert 3 notice by the Department of Health on the advice of the Health Protection Agency.^28^ Given the historical links between India and the UK, that the UK is the first western country to register the widespread presence of NDM-1-positive bacteria is unsurprising. However, it is not the only country affected. In addition to the first isolate from Sweden, a NDM-1-positive *K pneumoniae* isolate was recovered from a patient who was an Australian resident of Indian origin and had visited Punjab in late 2009. The isolate was highly resistant and carried *bla*_NDM-1_ on an incompatibility A/C type plasmid similar to those in India and the UK.

Several of the UK source patients had undergone elective, including cosmetic, surgery while visiting India or Pakistan. India also provides cosmetic surgery for other Europeans and Americans, and *bla*_NDM-1_ will likely spread worldwide. It is disturbing, in context, to read calls in the popular press for UK patients to opt for corrective surgery in India with the aim of saving the NHS money.^29^ As our data show, such a proposal might ultimately cost the NHS substantially more than the short-term saving and we would strongly advise against such proposals. The potential for wider international spread of producers and for NDM-1-encoding plasmids to become endemic worldwide, are clear and frightening.
